# A Rare Case of Hypokalemic Periodic Paralysis With Acute Urinary Retention: Diagnosis and Management

**DOI:** 10.7759/cureus.52839

**Published:** 2024-01-24

**Authors:** S Sara, Dhigvijay TV, Gokulesh DG, Balaji Elumalai, Mohamed Javid

**Affiliations:** 1 Internal Medicine, Public Health Centre, Chennai, IND; 2 General Surgery, Sri Saraswathi Hospital and Surgical Center, Krishnagiri, IND; 3 Internal Medicine, Madras Medical College, Chennai, IND; 4 Nephrology, Madras Medical College, Chennai, IND; 5 Urology, Chengalpattu Medical College, Chennai, IND

**Keywords:** flaccid paralysis, channelopathy, urinary retention, hypokalemia, hypokalemic periodic paralysis

## Abstract

Hypokalemic periodic paralysis (hypoPP) is a rare channelopathy caused by mutations in skeletal muscle ion channels that usually occurs in young individuals and adolescents. The etiology can be attributed to various factors, such as idiopathic or secondary causes. It is characterized by episodes of sudden flaccid muscle weakness. Timely detection may mitigate the risk of severe complications. Secondary causes of hypoPP, such as hyperthyroidism, should be ruled out, as this could lead to thyrotoxic periodic paralysis.

We report the case of a 19-year-old boy who presented to the ED with severe weakness in both the upper and lower extremities. The weakness rapidly progressed to his trunk and was accompanied by acute urinary retention. The physical examination was significant for bilateral upper and lower extremity weakness. Subsequent laboratory investigations revealed markedly low serum potassium levels. The patient’s symptoms resolved after the replacement of potassium, and he was discharged without neurological deficits. Although rarely accompanied by acute urinary retention, hypoPP must be differentiated from other causes of weakness and paralysis so that the proper treatment can be initiated quickly. The rarity of hypoPP, a condition seldom encountered in clinical practice, and the added rarity of its coexistence with acute urinary retention further underscore the uniqueness of this case report.

## Introduction

Hypokalemic periodic paralysis (hypoPP) is a rare condition with a prevalence of 1 in 100,000 [1,2]. It is an autosomal dominant disease that is usually seen in childhood or adolescence [3]. After 40 years of age, the episodes occur infrequently [4]. HypoPP was first described by Musgrave et al. [5,6] in 1727, and later, Jurkat-Rott et al. [7] and Ptáĉek et al. [8] reported the first disease-causing mutation in the CACNA1S gene in 1994. HypoPP is of two types. Nearly 70% to 80% of HypoPP is due to mutations in the alpha subunits of the skeletal muscle encoding dihydropyridine-sensitive L-type calcium channel gene CACN1AS (hypoPP1), and the remaining 30% is due to mutations in the skeletal muscle sodium channel gene SCN4A (hypoPP2) [1,9,10]. Sex hormones may have diverse roles in the development of hypoPP, so they may help in the development of treatments for ion channelopathies [10]. The sudden attack of hypoPP episodes is linked more commonly with females than males [10]. In this disorder, hypokalemia is due to a sudden and large influx of potassium into the cells rather than the actual net loss of potassium [11].

## Case presentation

A 19-year-old boy with no known comorbidities presented to the emergency room with quadriparesis and anuria. The symptoms started one day prior to his admission. He had weakness in both upper and lower extremities involving his trunk, which was rapidly progressive in nature. There was no history of similar episodes in the past. Prior to this episode, the patient had been healthy, consumed a carbohydrate-heavy meal, and had no recent history of diarrhea, chest pain, nausea, vomiting, shortness of breath, or weight loss. The patient had no history of lower urinary tract symptoms in the past and explicitly denied any history of drug intake. His vitals remained within the normal range. Concurrently, the skin exhibited a cool and dry texture, while the oral mucosa was moist. During the pelvic examination, a palpable suprapubic mass was found. Cranial nerve assessment revealed grossly intact function, but there was evident flaccid paralysis involving bilateral proximal and distal muscles of the upper and lower extremities, with a power of 2/5. This rendered the patient unable to walk. His sensory system was intact, with no pain or paresthesia. However, deep tendon reflexes exhibited hyporeflexia with bilateral flexor plantar reflexes. The respiratory and ocular muscles were spared. Examination of other systems yielded unremarkable findings.

**Figure 1:**
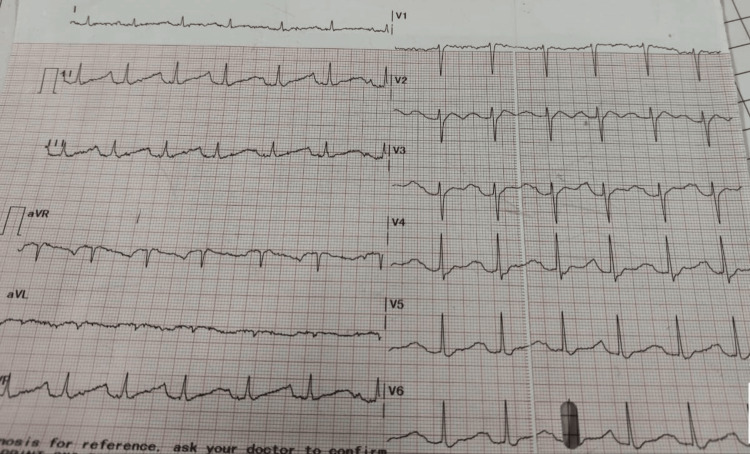
ECG with features suggestive of hypokalemia

The patient was admitted to the high dependency unit (HDU), and blood investigations revealed a significant reduction in serum potassium (2.5 mEq/L). The serum chloride (110 mEq/L) and sodium (144 mEq/L) were within the normal limits. The total count was 18560 cells/mm3, with neutrophils comprising 88% and leucocytes comprising 8.7%. Kidney function tests and other laboratory parameters were normal. The urinary retention was managed by inserting an 18 Fr urethral catheter immediately, and 450 ml of clear urine was drained. After initial stabilization and management of the patient, the urologist's opinion was sought. The suggested ultrasound of the abdomen and pelvis was performed, and it was normal. The 24-hour urine protein was within the normal range. Upon the neurologist's evaluation, an MRI of the brain and spinal cord was advised, which was suggestive of a posterior annular bulge in C5-C6, L5-S1 levels indenting the anterior thecal sac, lumbarization of the S1 vertebra, and Schmorl’s node in the D12 vertebra. The MRI was negative for any acute bleed or stroke. The patient demonstrated no red flag signs or symptoms for neural foramen narrowing or spinal cord compression. A thyroid function test (TFT) and an arterial blood gas (ABG) test were undertaken, and both were found to be within the normal limits. The ECG at the time of admission showed shallow T waves with prominent U waves consistent with hypokalemia (Figure 1).

The patient underwent treatment with intravenous potassium chloride. Subsequently, oral potassium supplements were initiated as the patient showed signs of recovery. He experienced no further complications during the period of his inpatient treatment. His serum potassium levels were normalized within 48 hours of initiating potassium replacement. Subsequently, a repeat ECG after two days was normal after the treatment. The patient’s urine output was closely monitored, and the patient started voiding normally once the urethral catheter was removed after two days. Serum potassium was measured daily due to the concern for rebound hyperkalemia. After the normalization of potassium, the patient’s power on both upper and lower extremities was 5/5, and he was able to walk again. The patient was discharged with oral potassium supplements. He was advised to avoid carbohydrate-rich meals and was followed up on an outpatient basis.

## Discussion

HypoPP is an uncommon condition marked by recurrent episodes of profound muscle weakness [5]. An episode of hypoPP is usually triggered by intense physical activity or by the consumption of a high-carbohydrate diet [5]. HypoPP is classified as primary and secondary, with the secondary form being the more prevalent subtype [5]. In most cases, this condition is overlooked in the first place [12]. The diagnosis of these conditions necessitates a thorough analysis of the patient's medical history, with particular attention to the timing, duration, and distribution of the symptoms [13]. Recurrent episodes of paralysis can lead to significant morbidity and complications, influencing the patients' employment as well as impacting their social, physical, and mental well-being [14].

Weakness is a common but vague symptom in both neurological and non-neurological disorders [15]. In the day preceding or following an episode, the patient may encounter symptoms such as myalgia, paraesthesia, and excessive thirst. This can potentially lead to severe complications like respiratory failure and cardiac arrhythmias [1,11,13]. It may be triggered by strenuous physical activity, carbohydrate-rich meals, infections, and stress [5,12]. A similar case of hypoPP has been reported in a patient and attributed to increased carbohydrate intake [16]. Urinary retention may occur with other conditions causing acute or subacute paralysis, but it is infrequently associated with hypoPP [17].

Laboratory investigations can reveal hypokalemia. To exclude secondary causes of hypoPP, a TFT should be done to rule out thyrotoxic periodic paralysis [18]. Abnormalities in the ECG are quite common with metabolic disorders such as hypoPP. Sinus bradycardia, the appearance of flattened T-wave or ST-segment depression, and prominent U-waves may be seen in an ECG [5]. It is vital to emphasize that this condition is autosomal dominant in two-thirds, with a male preponderance [15]. Therefore, patients who are hoping to have children should receive genetic counseling [15]. Other diagnostic options include genetic testing, provocative testing, and electromyography (EMG) [18].

The confirmation of hypoPP relies on meeting the established consensus criteria [17]. This typically involves a proband with a history of muscle weakness coupled with reduced serum potassium levels below 3.5 mmol/L and/or identifying a heterozygous pathogenic variant in either CACNA1S or SCN4A [17]. It is important to note that approximately 30% of individuals do not exhibit identifiable pathogenic variants in these specified genes [17]. Table 1 illustrates the diagnostic criteria for hypoPP [17].

**Table 1:** Supportive diagnostic criteria for hypoPP HypoPP: Hypokalemic periodic paralysis

No.	Diagnostic criteria
1.	Two or more attacks of muscle weakness with documented serum potassium less than 3.5mEq/L OR
2.	One attack of muscle weakness in the proband, and one attack of weakness in one relative with documented serum potassium less than 3.5mEq/L in at least 1 attack OR
3.	Three out of six of the following clinical or laboratory features: (i) Onset in the first or second decade; (ii)* *attack duration (muscle weakness involving one or more limbs) more than two hours;* *(iii) positive triggers (such as high carbohydrate-rich meals, rest after exercise and stress); (iv) improvement with potassium intake; (v) positive family history or genetically confirmed skeletal calcium or sodium channel mutation; (vi)* *positive McManis long exercise test AND
4.	Exclusion of other causes of hypokalemia (renal dysfunction, thyroid dysfunction, renal tubular acidosis, diuretic and laxative abuse)

In accordance with the above diagnostic criteria, our patient fulfills points 3 and 4. Therefore, the patient's presentation aligns with multiple facets of the established diagnostic criteria for hypoPP [17]. The treatment approach should include both the management of acute attacks and the prevention of future attacks [12]. The mainstay of treatment in hypoPP is potassium replacement. Parenteral and oral potassium chloride correction is given according to its severity [12,19]. Rebound hyperkalemia commonly occurs during hypokalemic correction [19]. Therefore, continuous monitoring of cardiac rhythm and blood potassium levels is essential to maintaining serum potassium within normal limits [12]. Prophylactic usage of spironolactone and acetazolamide has been successful, while long-term potassium supplementation may be required [12]. One limitation in our evaluation was the unavailability of genetic testing within our hospital. However, despite this constraint, the patient met other diagnostic criteria for hypoPP, supporting the diagnosis within the available resources.

## Conclusions

Hypokalemic periodic paralysis is a rare condition, and the coexistence of acute urinary retention with hypoPP is exceptionally uncommon. As hypoPP is characterized by common symptoms, it is prudent to consider this condition while ruling out other diseases whose symptoms resemble hypoPP. This underscores the need for comprehensive evaluation and the crucial role of the multidisciplinary team in the management of this condition. An acute episode of hypoPP can simply be ruled out by measuring serum potassium levels. Neglecting the timely diagnosis and treatment of this condition may have serious, even fatal, consequences. Therefore, early identification and intervention are paramount for effective management and patient safety.
